# DNA Barcoding Reveals Limited Accuracy of Identifications Based on Folk Taxonomy

**DOI:** 10.1371/journal.pone.0084291

**Published:** 2014-01-08

**Authors:** Hugo J. de Boer, Abderrahim Ouarghidi, Gary Martin, Abdelaziz Abbad, Anneleen Kool

**Affiliations:** 1 Department of Organismal Biology, Evolutionary Biology Centre, Uppsala University, Uppsala, Sweden; 2 Naturalis Biodiversity Center, Leiden, The Netherlands; 3 Natural History Museum, University of Oslo, Oslo, Norway; 4 Faculty of Science Semlalia, Cadi Ayyad University, Marrakech, Morocco; 5 Global Diversity Foundation, Marrakech, Morocco; Biodiversity Insitute of Ontario - University of Guelph, Canada

## Abstract

**Background:**

The trade of plant roots as traditional medicine is an important source of income for many people around the world. Destructive harvesting practices threaten the existence of some plant species. Harvesters of medicinal roots identify the collected species according to their own folk taxonomies, but once the dried or powdered roots enter the chain of commercialization, accurate identification becomes more challenging.

**Methodology:**

A survey of morphological diversity among four root products traded in the medina of Marrakech was conducted. Fifty-one root samples were selected for molecular identification using DNA barcoding using three markers, *trn*H-*psb*A, *rpo*C1, and ITS. Sequences were searched using BLAST against a tailored reference database of Moroccan medicinal plants and their closest relatives submitted to NCBI GenBank.

**Principal Findings:**

Combining *psbA-trnH*, *rpoC1*, and ITS allowed the majority of the market samples to be identified to species level. Few of the species level barcoding identifications matched the scientific names given in the literature, including the most authoritative and widely cited pharmacopeia.

**Conclusions/Significance:**

The four root complexes selected from the medicinal plant products traded in Marrakech all comprise more than one species, but not those previously asserted. The findings have major implications for the monitoring of trade in endangered plant species as morphology-based species identifications alone may not be accurate. As a result, trade in certain species may be overestimated, whereas the commercialization of other species may not be recorded at all.

## Introduction

### 1.1 Molecular Identification

Molecular techniques provide a powerful tool for DNA sequence-based species identification [1]–[6]. Barcoding research has developed rapidly. Three of the main organizations that advance barcoding research are the International Barcode of Life (iBOL) that promotes the generation of reference barcodes, the Consortium for the Barcode of Life (CBOL) that is devoted to the development of barcoding as a global standard [7], and Barcode of Life Data Systems (BOLD), an online workbench that aids collection, management, analysis, and use of DNA barcodes [8].The mitochondrial gene CO1 was proposed as the standard barcode for all animals [9], and assessments have since shown that CO1 can be used to distinguish over 90% of species in most animal groups [10], [11].

Barcoding in plants has developed at a slower pace. Evolution of the mitochondrial genome in most plants is far too slow to enable species distinction [12], [13]. The plastid genome evolves more rapidly, and various plastid genes and non-coding regions have been proposed as barcodes [7], [12], [14]–[18]. To ensure accurate species identification and differentiation, chosen molecular barcodes ought to exhibit relatively rapid evolution, yet must be flanked by conserved regions that can function as universal primer binding sites for PCR reactions [15]. In plants, a single barcoding locus combining these two traits has not been found, so a combination of two or more plastid loci are currently required in most cases for successful and comprehensive species identification [16]. CBOL [7] has proposed *matK* and *rbcL* as a combined universal barcode for land plants, to be used in tandem with other markers for individual identification projects, such as *psbA-trnH*, *atpF–atpH*, *psbK–psbI* [7], and *trnL* [19]. The China Plant Barcode of Life (BOL) Group [20] advocates the use of *matK* and *rbcL* together with *psbA-trnH* and ITS, based on universality and species discrimination.

Molecular identification, DNA barcoding, and related methodological approaches are still in development, and are certainly not without practical or theoretical problems. Generation of reference sequence libraries from herbaria, the main resource for accurately identified and vouchered collections, is a major challenge as DNA in older material is often degraded and hard to amplify [21]. Recent advances in next generation sequencing, whole genome sequencing and specific-target enrichment are opening up new possibilities for reference library creation [21]–[23]. Most systematists agree that species are evolving metapopulation lineages, but delimiting species is often more problematic [24], especially when hybridization plays an important role in plant speciation [25], [26]. Difficulties in distinguishing between intra- and interspecific variation represent a widespread problem in both morphological species delimitation and DNA barcoding [18], [27], [28].

Methods for matching an unknown query sequence with a reference database are either based on sequence similarity [29], [30], tree-based criteria [31]–[33], or character-based methods [34]–[36]. A recent study on barcoding of recently diverged species [37] showed that similarity-based and diagnostic methods significantly outperform tree-based methods. However, sequence similarity methods require a decision on a threshold at which a sequence is considered to belong to a certain taxon, which can be somewhat subjective and may be applicable to certain taxa but not to others [35], [36]. Nonetheless, the success of any method used to assign sequences to a certain taxon is ultimately dependent on the taxonomic coverage of the reference database [5].

### 1.2. The Moroccan Herbal Pharmacopoeia and Root Complexes

Traditional medicine plays an important role in many North African societies [38]. The Moroccan city of Marrakech, situated at a crossroads of historical trade routes between the Sahara Desert, High Atlas Mountains and surrounding coastal plains, is one of the richest trade hubs of medicinal plant products in the Maghreb [5], [39].

Herbalists, one type of practitioner of Moroccan traditional medicine, conduct retail trade of herbal medicines. Marrakech herbalists stock a variety of plant parts and plant-derived products, sold either separately or in mixtures. A majority of these plant parts, 61%, are harvested from the wild by specialized collectors and reach the herbalists through middlemen and wholesalers [39]–[41]. An important part of the plant inventory of Moroccan herbalists consists of barks and roots, which typically possess few physical characteristics that enable accurate morphological identification. All herbalists are able to provide information about the local name of a plant product, its medicinal uses and origins, but this information may be imprecise, or insufficient for species identification purposes, especially considering that herbalists usually do not possess knowledge of medicinal plants in the wild [40].

Confirming the identity of a root sample bought from herbalists has so far presented a challenge. Previous research has shown that Marrakech herbalists have significantly more difficulty identifying roots, seeds, bark, and resins, than leaves, flowers, and whole plants [42]. In a recent study, substitution or confusion of medicinal roots occurred in 54.6% of the 33 most commonly sold roots, and was more prevalent among herbalists than collectors [39].

The identity of the plants sold in these markets has conservation as well as public health implications. The collection and trade of medicinal roots has a large impact on natural plant populations as harvesting of roots usually requires the whole plant to be dug up [43]. Rare or endangered species could inadvertently be collected if they are easily confused with more abundant relatives. Increasing demand for medicinal products may lead to local over-harvesting and extinction of otherwise non-threatened plant species. Misidentified collections may result in introduction of toxic, non-efficacious or otherwise unsuitable species to the market, with potential health risks to consumers [44], [45]. Reliable identification is essential to support conservation efforts and to allay health and safety concerns.

Molecular identification of Marrakech roots using a tailored reference database uploaded to GenBank is demonstrably feasible and accurate, especially using multiple markers combined [5]. Barcoding of the market root samples yielded significantly different identifications from those reported in the popular and scientific literature. These samples correspond to root complexes divided into various subtypes by herbalists according to their folk taxonomies [46]. The herbalists use Moroccan Arabic colloquial terms such as *mezyana* (‘good’) and *soulouk* (‘inferior quality’) to differentiate better-quality and lesser-quality root subtypes in these complexes, according to morphological criteria.

The root products known locally as *amssekhsser*, *kelkh*, *tiguendizt* and *ziyata* were found to be subject to frequent substitution [39]. These products are all commonly traded in Marrakech, mostly sold as dried roots in 2–8 cm long fragments for prices that vary by product and store.

*Amssekhsser*, or *mserser*, which translates from Moroccan colloquial Arabic as ‘vertebrae wood’, are vernacular names usually attributed to *Polygonum aviculare* L., *Polygonum equisetiforme* Sm. and *Polygonum maritimum* L. [40]. The resemblance of the roots to a spinal cord forms the basis of its use in magic and fumigations to undermine a man's strength and force him to grovel before his mistress [40]. However, Ouarghidi *et al.* [39] identified samples of this product sold in the Marrakech markets as *Ammoides pusilla* (Brot.) Beistr., with *Apium nodiflorum* (L.) Lag. as a common substitute. Bellakhdar [40] also mentions that herbalists in Fes apply the name to *Daucus crinitus* Desf.

*Kelkh*, *lkleh*, or *l-kelha*, *l-kelh*, are variations of the vernacular name for *Ferula communis* L. all over the Arab world [40], [47]. *F. communis* is the source of a resin (*fassoukh*) exuded from the roots, and has been reported as a traditional medicine by Dioscorides, Galen, and Pliny [38], [40]. *Kelkh* is usually traded as a dry root or resin in the Marrakech herbal market, and herbalists report that the root is sometimes substituted with another product identified in the literature as *Thapsia villosa* L. [39].

Ziyata, or ziyyata, zayta, are a set of names associated with several different plant species: *Limoniastrum guyonianum* Durieu ex Boiss., *Limoniastrum ifniense* (Caball.) Font Quer, *Conium maculatum* L., *Polygonum maritimum* L., *Kundmannia sicula* (L.) DC., and *Apium nodiflorum* [40]. The root is used in fumigations to treat swellings, uterine bleeding, and colonitis [40]. Ouarghidi *et al.* [39] identified samples from the Marrakech markets as *Apium nodiflorum*, and did not find the reported true *Limoniastrum guyonianum*.

The Arabic term ‘aqirqarha and the Amazigh vernacular names tiguendizt, or tigentast, igentas, gentus are associated with *Anacyclus pyrethrum* (L.) Lag. [39], [40], [47], a species known in English as pellitory [48]. Pellitory roots are used widely in traditional medicine as a sternutatory, sialagogue, diaphoretic, and to treat liver diseases and toothache. The roots are reported to be substituted by the common *Armeria alliacea* (Cav.) Hoffmanns. & Link, or *Meum athamanticum* Jacq. in the markets of Marrakech [39].

This study investigates these four root complexes and tests the hypotheses that (1) molecular barcoding offers greater accuracy in identification of root material than morphology-based folk taxonomies and reports of correspondence between vernacular and scientific names in the literature; and (2) the presence of higher quality and lower quality root subtypes in these complexes is related to the inclusion of more expensive expected ingredients and cheaper substitutes, respectively.

## Results

### 2.1 PCR and sequencing rates and BLAST matching

DNA extraction worked for 87% of the root market samples. Only seven samples failed to yield PCR products. Sequencing success rates for the three loci, *psbA-trnH*, *rpoC1*, and ITS, were 77, 87 and 51%, and most roots were successfully sequenced for at least two of the regions (Data S1). A total of five ITS sequences obtained from the market samples turned out to be fungal contaminations.

All root sequences were queried in GenBank using BLAST, and the most probable corresponding species are compiled in Table 1 and Data S1. The identification success was dependent on the marker, the number of available markers and the sample. A total of 43 out of 47 samples were identified to species level, i.e. an unambiguous identification with a BLAST e-value of 0. Those samples for which three markers were available gave 10 species-level identifications; for two markers, 1 family, 1 genus and 27 species-level; and for 1 marker only, 2 genus and 6 species-level results. For the four samples that were not identified to species level the marker *rpoC1* was available in 100%, *trnH-psbA* in 50%, and ITS in 0% of cases. Species-level identification was possible with two or more markers in 90% of all root samples.

**Table 1:** Root complexes and their putative identifications based on folk taxonomies, and species identified from market samples of these complexes using molecular barcoding.

Amssekhsser
*Polygonum aviculare*
*Polygonum equisetiforme*
*Polygonum maritimum*
*Ammoides pusilla*
*Apium nodiflorum*
*Daucus crinitus*

Barcoding	Freq^1^	2%
*Daucus crinitus*	12	60
*Thapsia transtagana*	4	20
*Foeniculum vulgare*	1	5
Failed	3	15

Kelkh
*Ferula communis*
*Thapsia villosa*

Barcoding	Freq	%
*Thapsia platycarpa*	2	29
*Thapsia transtagana*	1	14
*Thapsia* sp.	1	14
Failed	3	43

Tiguendizt
*Anacyclus pyrethrum*
*Armeria alliacea*
*Meum athamanticum*

Barcoding	Freq	%
*Anacyclus valentinus*	16	80
*Anacyclus* sp.	1	5
*Plantago* sp.	1	5
*Scorzonera caespitosa*	1	5
*Carlina brachylepis*	1	5

Zziyata
*Limoniastrum guyonianum*
*Limoniastrum ifniense*
*Conium maculatum*
*Polygonum maritimum*
*Kundmannia sicula*
*Apium nodiflorum*

Barcoding	Freq	%
*Daucus crinitus*	3	43
*Kundmannia sicula*	2	29
Apiaceae indet.	1	14
Failed	1	14

Barcoding	Freq^1^	%^2^
*Daucus crinitus*	12	60
*Thapsia transtagana*	4	20
*Foeniculum vulgare*	1	5
Failed	3	15

^1^ Frequency of identifications to this species within complex.

^2^ Percentage of root products in complex identified to this species.

### 2.3 Molecular identification, the herbal pharmacopeia, and local qualities

The identifications derived from molecular methods of all but two of the samples of the *amssekhsser*, *kelkh*, *tiguendizt* and *ziyata* root complexes were different from those obtained based on interviews with herbalists and consultation of the standard pharmacopeia and other literature sources (Table 1 and Data S1). Only two of the molecular identifications matched an expected species for the four complexes, and only 32% matched with species in an expected genus.

Seventeen of 20 *amssekhsser* market samples yielded sequences for one or more of the three barcoding markers (Table 1). The majority, 71%, matched GenBank sequences of *Daucus crinitus* (Apiaceae), whereas another 24% matched with *Thapsia transtagana* Brot. (Apiaceae). Four of seven *kelkh* market samples yielded sequences (Table 1), and all samples were identified as *Thapsia* spp. (Apiaceae). All twenty *tiguendizt* samples yielded sequences, and 85% were identified as species of *Anacyclus* (Asteraceae), and the remainder as other species in Asteraceae or in Plantaginaceae. Six of seven *ziyata* samples yielded sequences, and two samples matched the expected species, *Kundmannia sicula*. Three samples were identified as *D. crinitus*, and one had ambiguous identification results, and could not be identified with certainty beyond Apiaceae.

Herbalists classified all samples after purchase in two different categories, *mezyana* – the true remedy – or *soulouk*, a substitute (Data S2). The herbalists treat the species within a complex as subtypes of the same vernacular name implying they are perceived to share the same medicinal properties and are used to treat the same ailments. The likely intention is to substitute the true remedy with a similar one in terms of use and morphology, and suggests that the herbalist can consistently distinguish the subtypes.

## Discussion

### 3.1 Complexes and label species

Linares and Bye [49] studied species complexes in Mexico, and found that although a complex can consist of many species, it usually includes one most salient species, which is traded most commonly and far beyond the geographical range where it grows naturally or is cultivated. These salient species were termed ‘label’ species, a concept that applies to the complexes studied. According to barcoding results reported here, the label species of *amssekhsser* and *ziyata* is *Daucus crinitus*; that of *kelkh Thapsia* spp.; and that of tiguendizt *Anacyclus valentinus* L. All the label species differ from those previously asserted by morphological studies [39], [40], [47], [50], [51]. This shift can be the result of gradual substitution due to overharvesting and resulting scarcity [5], [39], a lack of accuracy in morphology-based methods for studying root complexes, or differences in sampling strategy among the various studies. Further clarity in the identification of roots traded in markets could be achieved by tracing products from the market to the primary collector and locating and documenting differences in classification along the market value chain.

### 3.2 Amssekhsser

Bellakhdar [40] asserts that *amssekhsser* corresponds to roots of different species of *Polygonum* (Polygonaceae), but also mentions that herbalists in Fes apply the name to *Daucus crinitus*. DNA barcoding revealed that market samples from Marrakech consist of only three species, *D. crinitus*, *Thapsia transtagana*, and *Foeniculum vulgare* Mill., with a clear majority being *D. crinitus* (71%; Table 1). All three species belong to the Apiaceae, a family with uniform root morphology [49], facilitating undetected substitution. Ouarghidi *et al.* [39], using interviews and herbarium vouchers made with collectors, identified this product as *Ammoides pusilla*, with *Apium nodiflorum* and a product called *deryass* as common substitutes. The *deryass* substitution could explain the identification of *Thapsia transtagana* in four (24%) of the samples, as this complex is identified in the literature as *Thapsia transtagana*, *T. garganica* L., or *T. villosa* L.. Molecular identification of *D. crinitus* in 71% of the tested market samples illustrates the shortcomings of morphology-based ethnobotanical studies, as none had previously identified this species from the markets of Marrakech [39], [40], [47], [50], [51].

### 3.2 Kelkh

Previous literature [40], [47] mentioned that *kelkh* is the vernacular name used for *Ferula communis*, and that it is the source of *fassoukh* resin. Herbalists and wholesalers mentioned in interviews that the sale of true *kelkh*, presumably the root of *F. communis*, has declined since the 1980s, even though the resin is still widely available. The molecular identifications showed that all sampled roots traded as *kelkh* belong to the genus *Thapsia* (Apiaceae). These identifications match findings by Ouarghidi *et al.* [39], which show that *kelkh* is sometimes substituted with *deryass*. The local under-differentiation [46] of *deryass* can be explained by the morphological similarity of the roots of these species, which are all horizontally streaked and have hairy tufts at the stem bases [52]. Identification tasks by herbalists show that confusion and substitution in *kelkh* is common [39], and could result from its relatively low level of trade. The reduction in trade has probably led to loss of knowledge of its use, as well as to misidentification and unavailability in the wholesalers markets. All the species discussed, F. *communis*, *T. villosa*, *T. platycarpa* and *T. garganica*, are common in Morocco [53]–[57].

### 3.3 Tiguendizt

Molecular identification of the 20 *tiguendizt* samples showed the majority to be *Anacyclus valentinus*, a species found commonly in Morocco, but not previously suggested as the source of this product. All previous studies suggested that *tiguendizt* was *Anacyclus pyrethrum* [39], [40], [47], or a number of different substitutes, including *Armeria alliacea* [39], *Meum athamanticum* [39], or *Catananche caespitosa* Desf. [5]. The barcoded samples in this study add to the number of known substitutes, with *Plantago* sp., *Scorzonera caespitosa* Pomel, and *Carlina brachylepis* (Batt.) Meusel & Kästner (Table 1). The principal finding remains that *A. valentinus* is an important source of *tiguendizt* sold in Marrakech markets instead of *A. pyrethrum*. Both species occur in Morocco, and are commonly found on disturbed ground in the rocky and sandy soils of habitats from 800–3000 m in the Middle, High and Anti-Atlas mountains [58]. Humphries, in a revision of the genus [58], suggests that *A. valentinus* might be a hybrid between *A. homogamos* (Maire) Humphries and *A. radiatus* Loisel, but there is no molecular data to support this assertion. Barcoding identification of hybrid species poses a challenge as DNA from both parent species could be present in the hybrid populations [59].

### 3.4 Ziyata

Morphology-based identification of plant products known as *ziyata* has pointed to species belonging to Apiaceae, Plumbaginaceae, and Polygonaceae [39], [40], [47]. Two samples identified through barcoding were *Kundmannia sicula*, one of three Apiaceae species suggested for this complex (Table 1). Three other samples were identified as *Daucus crinitus*, a species common in the High Atlas mountains south of Marrakech. Ouarghidi *et al.* [39] cite herbalists who report that *ziyata* is used as a substitute for *amssekhsser*, and this is partly supported by the molecular identification which shows that both complexes include *D. crinitus* roots. During interviews, herbalists reported that *ziyata* is toxic, causes acute oral inflammation and should not be ingested. However, the two species identified using DNA barcoding, *D. crinitus* and *K. sicula*, are both known to be non-toxic. Analysis of additional market samples of *ziyata* could provide insight into other species commercialized under this name, and possibly help identify the source of the reported toxicity in this complex.

### 3.5 Reported qualities and substitution

It is common for herbalists to distinguish between samples that are *mezyana* – the true remedy – and *soulouk*, a substitute (Data S2). Herbalists treat categories within a complex as subtypes of the same vernacular name implying that these are perceived to share the same medicinal properties and are used to treat the same ailments. Our hypothesis that quality of the root subtypes in these complexes is related to the inclusion of more expensive expected ingredients and cheaper substitutes, was hard to test because although all comprise more than one species, none were those that were previously asserted. In the complex *amssekhsser*, *mezyana* is applied mainly to *D. crinitus* (84.6% of sequenced samples) and soulouk mainly to *T. transtagana* (75%). The label species of *amssekhsser* is *D. crinitus*, and the correspondence between the qualities and these species suggests a reliable folk classification. The complexes *kelkh* and *ziyata* each have too few samples to study perceived quality and species identification (Data S2). In *tiguendizt* herbalists distinguish two subtypes, *meyzana* with a thick root, a yellow-brownish color and an anesthetizing taste, and *soulouk* with a thin root. *Mezyana* is applied only to *A. valentinus* (100% of sequenced samples), but so is *soulouk* (73.3% of *soulouk* denoted samples are *A. valentinus*). That ten of the *soulouk* samples and six of the *mezyana* samples barcoded in this study were *A. valentinus*, suggests that herbalists over-differentiate this species, that is, their classification recognizes morphological and other differences that are not taken into account in scientific categorization. Ouarghidi *et al.* [39] reported that some *tiguendizt* collectors differentiate between *iguendez* and *tiguendizt*, with the latter being of inferior quality. In that study, herbarium collections of *iguendez* were identified as *Anacyclus pyrethrum* (L.) Link var. *pyrethrum* and *tiguendizt* as *Anacyclus pyrethrum* var. *depressus* (Ball) Maire. The specimens that were used to obtain the reference sequences in this study for the alternative species and varieties *A. pyrethrum* var. *pyrethrum*, *A. pyrethrum* var. *depressus*, *A. homogamos*, *A. monanthos*, *A. radiatus* were all identified by specialists working on the flora of Morocco at the University of Reading herbarium, known for its extensive collections of the Moroccan flora. The incongruence in identifications between [39] and this study highlight the difficulty of morphological identification of *Anacyclus*.

### 3.6 Conclusion

The four root complexes, as analyzed through barcoding of plant products traded in Marrakech markets, all comprise more than one species, but not those previously asserted. Molecular barcoding and folk classifications differ in their ability to differentiate and identify variation. Folk classifications can both under-differentiate, as in the case of *kelkh*, in which different *Thapsia* species are used indiscriminately; and over-differentiate, as in *tiguendizt*, in which the local categories *mezyana* and *soulouk* apparently differentiate subtypes within *A. valentinus*. Molecular barcoding always yields a species of highest probability, but is limited by the sequence reference library through which the identifications are made.

Each complex studied here consists of multiple identified species, but each complex has a single species that is more salient than others. The label species of *amssekhsser* and *ziyata* is *D. crinitus*; that of *kelkh Thapsia* spp.; and that of *tiguendizt A. valentinus*, but all label species differ from those previously asserted by folk classification and morphological studies [39], [40], [47], [50], [51]. A relation between high quality - *mezyana* - and inferior quality - *soulouk*- was only found in *amssekhsser*, where *mezyana* was applied mainly to the label species, and *soulouk* mainly to other species. In the complex *tiguendizt* both *mezyana* and *soulouk* were applied to the label species. These results provide insight into the species composition of medicinal products that are currently traded under a specific vernacular name. Species substitution can lead to gradual change in species complexes, and it cannot be ruled out that the previously asserted species were formerly traded under these names in Marrakech or continue to be marketed elsewhere in Morocco today. Similarly, future change in availability due to harvesting, exploitation or invasive species could lead to further label species variation in these complexes. The findings have major implications for the monitoring of trade in endangered plant species, as morphological identification of marketed plant products by itself may not be accurate. As a result, trade in certain species may be overestimated, whereas the commercialization of other species may not be documented at all.

## Materials and Methods

### 4.1 Herbalists and Market Samples

Market samples were purchased from three different herbal markets in the city of Marrakech, each characterized differently: 1) the Mellah, known for wholesale trade of plant products; 2) the Rahba Lakdima, where retailers sell single ingredients, mixtures and spices to both locals and tourists; and 3) individual herbalist stores scattered throughout the old town (*medina*) and the new city (*nouvelle ville*) of Marrakech selling mainly to locals. A total of 54 market samples of medicinal roots were purchased from a total of 32 herbalists from September 2008 to September 2010. Prior informed consent was obtained from all herbalists, and participating herbalists were informed about the methods and objectives of the project. All participation was voluntary. The vernacular name for each sample given by the herbalist was recorded, along with the herbalist's name and the place and date of purchase. Open interviews about the root complexes were carried out in parallel to purchasing samples. Herbalists were assigned codes to protect their privacy (Data S2). All roots were purchased as single products to avoid mixtures of different plants in the samples, which are stored in the collections of the Natural History Museum of Marrakech. A memorandum of understanding and material transfer agreement between Cadi Ayyad University, Morocco, and Uppsala University, Sweden, regulated transfer of material for DNA extraction.

### 4.2 Reference Database

The plant barcoding reference database of southern Morocco medicinal roots created by Kool *et al.* [5] was used in combination with voucher-referenced sequences deposited in GenBank. The Kool et al. [5] data has been deposited in BOLD and the associated records in GenBank are now linked and managed from BOLD (Data S3). The reference collection was based on species known to occur in Morocco [53]–[57], [60], [61], as this is the main origin of medicinal roots traded in Marrakech [50]. All species identified in the literature as putative candidates for the root complex species, were present in the reference set used for BLAST matching, with the exception of *Limoniastum ifniense* (Caball.) Font Quer for which a closely related species was present, *L. guyonianum* Durieu ex Boiss (see Table 1, species listed directly under complex name). Those species identified using BLAST matching with the reference set were present including additionally multiple closely related species within the same genus, collected in Morocco (Table 1, species listed under Barcoding). This includes 5 species in the genus *Anacyclus*, 5 in *Carlina*, 6 in *Daucus*, 4 in *Thapsia*, and the one species each in the monotypic genera *Foeniculum* and *Kundmannia*, cf [5] and Data S2.

### 4.3 DNA Extraction, PCR and Sequencing

Root material was extracted using a slightly modified version of the Carlson/Yoon DNA isolation procedure [62] as described in [5]. Each total DNA extract was further purified using the GE Illustra GFX™ PCR DNA and Gel Band Purification Kit following the manufacturer's protocol (GE Healthcare).

Barcoding loci and primers were selected from the Royal Botanic Gardens Kew Phase 2 Protocols and Update on plant DNA barcoding [63] to match with [5]. These consisted of ITS primers *ITS-4* [64] and *ITS-5* [65], matK primers, *matK-2.1a* and *matK-5* [63], *rpoC1* primers, *rpoC1-2* and *rpoC1-4* [63], and *psbA-trnH* primers, *psbA* and *trnH* [66]. PCR amplification of ITS, *matK*, *rpoC1* and *psbA-trnH* was conducted on purified total DNA from all market samples following the protocols described in [5]. Sequencing was performed by Macrogen Inc. (Seoul, South Korea) on an ABI3730XL automated sequencer (Applied Biosystems). The same primers used in PCR amplification were also used for the sequencing reactions. Trace files were aligned with the programs Gap4 and Pregap4 [67], both modules in the Staden package [68]. All sequences were submitted to BOLD (Data S1).

### 4.4 Data Analyses

Based on studies showing that BLAST outperforms other molecular identification methods [5], [20], [69], [70], NCBI's web-based megablast algorithm using the default settings was used to identify the query sequences. Each identification was made manually, taking E-value, maximum identity, number of closely related species represented in the database, as well as distribution of the plant(s) in question into consideration.

## Supporting Information

Data S1 **Genbank BLAST identification of root product complexes and associated BOLD identification numbers.**
(XLSX)

Data S2 **Overview of Amssekhsser, Kelkh, Tiguendizt and Zziyata root samples.**
(XLSX)

Data S3 **Morocco reference database Kool et al. 2012 PLOS ONE with Genbank accession numbers and matching BOLD-IDs.**
(XLSX)
